# A multivariate statistical test for differential expression analysis

**DOI:** 10.1038/s41598-022-12246-w

**Published:** 2022-05-18

**Authors:** Michele Tumminello, Giorgio Bertolazzi, Gianluca Sottile, Nicolina Sciaraffa, Walter Arancio, Claudia Coronnello

**Affiliations:** 1 Department of Economics, Business and Statistics, University of Palermo, Palermo, Italy; 2 Institute for Biomedical Research and Innovation, National Research Council, Palermo, Italy; 3 Advanced Data Analysis Group, Fondazione Ri.MED, Palermo, Italy

**Keywords:** Biological techniques, Bioinformatics, Gene expression analysis, Software

## Abstract

Statistical tests of differential expression usually suffer from two problems. Firstly, their statistical power is often limited when applied to small and skewed data sets. Secondly, gene expression data are usually discretized by applying arbitrary criteria to limit the number of false positives. In this work, a new statistical test obtained from a convolution of multivariate hypergeometric distributions, the Hy-test, is proposed to address these issues. Hy-test has been carried out on transcriptomic data from breast and kidney cancer tissues, and it has been compared with other differential expression analysis methods. Hy-test allows implicit discretization of the expression profiles and is more selective in retrieving both differential expressed genes and terms of Gene Ontology. Hy-test can be adopted together with other tests to retrieve information that would remain hidden otherwise, e.g., terms of (1) cell cycle deregulation for breast cancer﻿ and (2) “programmed cell death” for kidney cancer.

## Introduction

Differential expression analysis (DEA) is a large-scale inference procedure used to identify genes whose expression differs under different biological conditions. Several variants of the t-test have been developed to perform DEA^1,2^. However, the small and skewed data typically analysed make the parametric assumptions rarely satisfied and, therefore, t-test *p*-values are often unreliable^3^. The easiest solution to small data size would be to increase the number of experiments, which, however, would increase experimental costs accordingly. Furthermore, data collected for poorly expressed genes are characterized by several zeros in the data. This evidence violates the typical assumptions under which t-test statistics are reliable. As a result, t-tests tend to increase type I errors and overestimate the number of significant genes. Alternative definitions of the t-test have been proposed to reduce the impact of small samples and low expression variability, e.g., moderated t-test^4^ and Significance Analysis of Microarray (SAM)^5^. Indeed, we compare the performance of the proposed test for differential expression with the one of moderated t-test and SAM. Conversely, t-tests applied to large data sets also produce too many significant genes; this depends on the fact that average expression differences may be significantly different from zero from a statistical point of view﻿ but are not large enough to be biologically meaningful.

A common strategy to reduce the number of selected differentially expressed genes is to discretize the gene expression. The discretization of gene expression data (GED) is widely used in genomics analysis. Despite a certain loss of information, GED discretization is often used as a preprocessing step to reduce raw data noise and facilitate the interpretation of data^6^. Several algorithms require data discretization during the preprocessing, e.g., the biclustering method^7^. Moreover, many network models require discrete data as input, e.g., Bayesian Networks﻿ and logical networks^8,9^. Despite the importance of discretization in transcriptomics, the criteria behind discretization methods are always arbitrary: the log2-Fold Change (FC)-discretization^10^ depends on an arbitrary set threshold, usually equal to 1, 1.5 or 2; the Equal Width discretization^11^ depends on a tuning parameter; a simple rank-based discretization depends on the Xth percentile that identifies the top-X% genes.

We propose a novel statistical test for DEA based on a convolution of multivariate hypergeometric distributions (Hy-test), which addresses both issues of t-test methods discussed before. Moreover, the method implicitly comprises a novel approach for data discretization, which is free from arbitrary parameters. At the price of a slight loss of information, Hy-test presents the following advantages with respect to the currently used methods:

1. It is free from parametric assumptions;
2. It allows implicitly provides a discretization of the expression profiles;
3. It is more conservative than the t-tests, reducing type I errors.
4. It can be integrated with other methods.

In this paper, the Hy-test has been applied to investigate breast and kidney cancer tissues, and results have been compared to those obtained through the t-test approach. The results indicate that the joint use of the Hy-test and moderated t-test allows one to understand the biological implications of DEA better.

## Methods

### Algorithm

Let's consider a gene expression profile recorded in two experimental conditions, e.g., normal and cancer tissues, for n pairs of tissues. We estimate a threshold couple able to discretize gene expression as “downregulated”, “upregulated”, and “no-changed”. The optimum thresholds are obtained by maximizing the disagreement between the discretized levels of the two different experimental conditions. Applying the thresholds $k_{1} ,k_{2}$ on the whole expression of a single gene, we obtain two discretized vectors, one for healthy tissues, say $\vec{v}_{H}$, and one for diseased tissues, say $\vec{v}_{D}$, with entries that take values {-1,0,1}, which means “downregulated”, “no-changed”, and “upregulated”, respectively. The thresholds $k_{1} ,k_{2}$ are estimated by maximizing the quantity

1 $$H\left( {\vec{v}_{H} ,\vec{v}_{D} } \right) = n_{ + , - } + n_{ - , + }$$

where $n_{ + , - } \left( {n_{ - , + } } \right)$ is the number of tissue couples that present upregulated normal (cancer) tissues paired with downregulated cancer (normal) tissues. Optimization research has been carried out by using a genetic algorithm^12^. A threshold has been estimated for each gene of the dataset. However, this method can also be easily adapted to extract a single cut-off couple for all genes.

As soon as optimal values for the thresholds, $k_{1} {\text{and }}k_{2} ,{ }$ are determined, we calculate a *p*-value to assess if gene expression is significantly different between cancer and normal tissues. To associate a *p*-value with $H\left( {\vec{v}_{H} ,\vec{v}_{D} } \right)$ it’s necessary, as a preliminary step, to evaluate the probability that a value of $H\left( {\vec{v}_{H} ,\vec{v}_{D} } \right) = n_{ + , - } + n_{ - , + }$ occurs by chance. For the sake of readability, we describe the analysis in two steps. In the first one, we set constraints on the total number of positive, negative, and null signs on both vectors in the null hypothesis, then we describe the distribution of the null model after relaxing these constraints. Specifically, in the first step, the null model depends on the external parameters ${\vec{\text{K}}}_{{\text{H}}} = \left( {K_{H}^{ + } ,K_{H}^{ - } ,K_{H}^{0} } \right)$ and $\vec{K}_{{\text{D}}} = \left( {K_{D}^{ + } ,K_{D}^{ - } ,K_{D}^{0} } \right)$, where $K_{H}^{i} \left( {K_{D}^{i} } \right)$ is the total number of tissues with sign I in vector $\vec{v}_{H} \left( {\vec{v}_{D} } \right)$ with, $i$ in {-1.1,0}. Such parameters are not independent. Indeed $K_{H}^{ + } + K_{H}^{ - } + K_{H}^{0} = K_{D}^{ + } + K_{D}^{ - } + K_{D}^{0} = n$, where $n$ is the total number of tissue couples in the dataset. We are interested in calculating the probability that matrix

2 $$C = { }\left( {\begin{array}{*{20}c} {n_{ + , + } } & {n_{ + , - } } & {n_{ + ,0} } \\ {n_{ - , + } } & {n_{ - , - } } & {n_{ - ,0} } \\ {n_{0, + } } & {n_{0, - } } & {n_{0,0} } \\ \end{array} } \right)$$

occurs by chance, subject to the aforementioned constraints. An entry $n_{i,j}$ of $C$ represents the number of tissues that display sign $i$ in vector $\vec{v}_{H}$ and sign $j$ in $\vec{v}_{D}$. Notation $C$ is used here because sometimes matrices such as the one above are indicated as “confusion" matrices. Entries of matrix $C$ are not independent due to the constraints on the number of positive, negative, and null signs described above. Specifically, they are linearly dependent according to the following six equations:

3 $$\left\{ {\begin{array}{*{20}c} {n_{ + , + } + n_{ + , - } + n_{ + ,0} = K_{H}^{ + } } \\ {n_{ - , + } + n_{ - , - } + n_{ - ,0} = K_{H}^{ - } } \\ {n_{0, + } + n_{0, - } + n_{0,0} = K_{H}^{0} } \\ {n_{ + , + } + n_{ - , + } + n_{0, + } = K_{D}^{ + } } \\ {n_{ + , - } + n_{ - , - } + n_{0, - } = K_{D}^{ - } } \\ {n_{ + ,0} + n_{ - ,0} + n_{0,0} = K_{D}^{0} } \\ \end{array} } \right.$$

This linear system has rank equal to 5, because of the linear relationship between parameters: $K_{H}^{ + } + K_{H}^{ - } + K_{H}^{0} = K_{D}^{ + } + K_{D}^{ - } + K_{D}^{0} = n$. Therefore, it can be solved as

4 $$\left\{ {\begin{array}{*{20}l} {n_{ + ,0} = K_{H}^{ + } - n_{ + , - } + n_{ + , + } } \hfill \\ {n_{ - ,0} = K_{H}^{ - } - n_{ - , - } - n_{ - , + } } \hfill \\ {n_{0, + } = K_{D}^{ + } - n_{ - , + } + n_{ + , + } } \hfill \\ {n_{0, - } = K_{D}^{ - } - n_{ - , - } - n_{ + , - } } \hfill \\ {n_{0,0} = K_{H}^{0} + K_{D}^{0} - n + n_{ - , - } + n_{ - , + } + n_{ + , - } + n_{ + , + } } \hfill \\ \end{array} } \right.$$

This result indicates that matrix $C$ is fully determined by the knowledge of $n_{ - , - } ,n_{ - , + } ,n_{ + , - } ,{\text{ and }}n_{ + , + }$. Therefore, the probability

5 $$P\left( C \right) = P\left( {n_{ - , - } ,n_{ - , + } ,n_{ + , - } ,n_{ + , + } {|}\vec{H}_{H} ,\vec{K}_{D} } \right) = { } = { }P\left( {n_{ - , - } ,n_{ - , + } |n_{ + , - } ,n_{ + , + } ,\vec{K}_{H} ,\vec{K}_{D} } \right)P(n_{ + , - } ,n_{ + , + } |\vec{K}_{H} ,\vec{K}_{D} )$$

where according to a simple combinatorial analysis of the problem,

6 $$P\left( {n_{ + , - } ,n_{ + , + } {|}\vec{K}_{H} ,\vec{K}_{D} } \right) = \frac{{\left( {\begin{array}{*{20}c} {K_{D}^{ + } } \\ {n_{ + , + } } \\ \end{array} } \right)\left( {\begin{array}{*{20}c} {K_{D}^{ - } } \\ {n_{ + , - } } \\ \end{array} } \right)\left( {\begin{array}{*{20}c} {K_{D}^{0} } \\ {n_{ + ,0} } \\ \end{array} } \right)}}{{\left( {\begin{array}{*{20}c} n \\ {K_{H}^{ + } } \\ \end{array} } \right)}}$$

and

7 $$P\left( {n_{ - , - } ,n_{ - , + } {|}n_{ + , - } ,n_{ + , + } ,\vec{K}_{H} ,\vec{K}_{D} } \right) = \frac{{\left( {\begin{array}{*{20}c} {K_{D}^{ + } - n_{ + , + } } \\ {n_{ - , + } } \\ \end{array} } \right)\left( {\begin{array}{*{20}c} {K_{D}^{ - } - n_{ + , - } } \\ {n_{ - , - } } \\ \end{array} } \right)\left( {\begin{array}{*{20}c} {K_{D}^{0} - n_{ + ,0} } \\ {n_{ - ,0} } \\ \end{array} } \right)}}{{\left( {\begin{array}{*{20}c} {n - K_{H}^{ + } } \\ {K_{H}^{ - } } \\ \end{array} } \right)}}$$

The distribution of $C$ allows to calculate the probability

8 $$P\left[ {H\left( {\vec{v}_{H} ,\vec{v}_{D} } \right) = x} \right] = P\left( {n_{ + , - } + n_{ - , + } = x} \right) = P\left( x \right)$$

As

$$\begin{aligned} P\left( x \right) = & \mathop \sum \limits_{{n_{ + , + } ,n_{ - , - } ,n_{ - , + } }} P\left( {n_{ - , - } ,n_{ - . + } |x - n_{ - , + } ,n_{ + , + } ,\vec{K}_{H} ,\vec{K}_{D} } \right)P\left( {x - n_{ - , + } ,n_{ + , + } |\vec{K}_{H} ,\vec{K}_{D} } \right) \\ = & \mathop \sum \limits_{{\left\{ {n_{ + , + } ,n_{ - , - } ,n_{ - , + } } \right\}}} \frac{{\left( {\begin{array}{*{20}c} {K_{D}^{ + } } \\ {n_{ + , + } } \\ \end{array} } \right)\left( {\begin{array}{*{20}c} {K_{D}^{ - } } \\ {x - n_{ - , + } } \\ \end{array} } \right)\left( {\begin{array}{*{20}c} {K_{D}^{0} } \\ {n_{ + ,0} } \\ \end{array} } \right)}}{{\left( {\begin{array}{*{20}c} n \\ {K_{H}^{ + } } \\ \end{array} } \right)}}\frac{{\left( {\begin{array}{*{20}c} {K_{D}^{ + } - n_{ + , + } } \\ {n_{ - , + } } \\ \end{array} } \right)\left( {\begin{array}{*{20}c} {K_{D}^{ - } - x + n_{ - , + } } \\ {n_{ - , - } } \\ \end{array} } \right)\left( {\begin{array}{*{20}c} {K_{D}^{0} - n_{ - ,0} } \\ {n_{ - ,0} } \\ \end{array} } \right)}}{{\left( {\begin{array}{*{20}c} {n - K_{H}^{ + } } \\ {K_{H}^{ - } } \\ \end{array} } \right)}} \\ \end{aligned}$$

According to this distribution, the p-value associated with an observation $\hat{x} = {\hat{\text{n}}}_{ - , + } + {\hat{\text{n}}}_{ + , - }$ is :

9 $$P\left( {x \ge \hat{x}} \right){ } { } = \mathop \sum \limits_{{\left\{ {n_{ + , + } ,n_{ - , - } ,n_{ - , + } ,x \ge \hat{x}} \right\}}} \frac{{\left( {\begin{array}{*{20}c} {K_{D}^{ + } } \\ {n_{ + , + } } \\ \end{array} } \right)\left( {\begin{array}{*{20}c} {K_{D}^{ - } } \\ {x - n_{ - , + } } \\ \end{array} } \right)\left( {\begin{array}{*{20}c} {K_{D}^{0} } \\ {n_{ + ,0} } \\ \end{array} } \right)}}{{\left( {\begin{array}{*{20}c} n \\ {K_{H}^{ + } } \\ \end{array} } \right)}}\frac{{\left( {\begin{array}{*{20}c} {K_{D}^{ + } - n_{ + , + } } \\ {n_{ - , + } } \\ \end{array} } \right)\left( {\begin{array}{*{20}c} {K_{D}^{ - } - x + n_{ - , + } } \\ {n_{ - , - } } \\ \end{array} } \right)\left( {\begin{array}{*{20}c} {K_{D}^{0} - n_{ - ,0} } \\ {n_{ - ,0} } \\ \end{array} } \right)}}{{\left( {\begin{array}{*{20}c} {n - K_{H}^{ + } } \\ {K_{H}^{ - } } \\ \end{array} } \right)}}$$

In the second step, we relax the constraints on the total number of positive, negative, and null signs in both the vectors associated with healthy (H) and diseased tissues (D). This is done by only assuming that the overall (across H and D tissues) number of positive, K+ , negative, K-, and null signs, K0, are set. In this case, we have to modify the previous formula. Specifically, let's indicate with $K^{ + } = K_{R}^{ + } + K_{G}^{ + } ,K^{ - } = K_{R}^{ - } + K_{G}^{ - }$ and $K^{0} = K_{R}^{0} + K_{G}^{0}$ the total number of positive, negative and null signs across the $2n = K^{ + } + K^{ - } + K^{0}$ samples, that is, two times the number of paired tissues. In this case, the null hypothesis is attained by assuming that $n$ tissues are randomly selected to be pathological, and paired with the others, which are supposed to be the healthy ones. Therefore:

10 $$P\left( {x \ge \hat{x}} \right) = = \mathop \sum \limits_{Q} \frac{{\left( {\begin{array}{*{20}c} {K^{ + } } \\ {K_{D}^{ + } } \\ \end{array} } \right)\left( {\begin{array}{*{20}c} {K^{ - } } \\ {K_{D}^{ - } } \\ \end{array} } \right)\left( {\begin{array}{*{20}c} {K^{0} } \\ {K_{D}^{0} } \\ \end{array} } \right)}}{{\left( {\begin{array}{*{20}c} {2n} \\ n \\ \end{array} } \right)}}\frac{{\left( {\begin{array}{*{20}c} {K_{D}^{ + } } \\ {n_{ + , + } } \\ \end{array} } \right)\left( {\begin{array}{*{20}c} {K_{D}^{ - } } \\ {x - n_{ - , + } } \\ \end{array} } \right)\left( {\begin{array}{*{20}c} {K_{D}^{0} } \\ {n_{ + ,0} } \\ \end{array} } \right)}}{{\left( {\begin{array}{*{20}c} n \\ {K_{H}^{ + } } \\ \end{array} } \right)}}\frac{{\left( {\begin{array}{*{20}c} {K_{D}^{ + } - n_{ + , + } } \\ {n_{ - , + } } \\ \end{array} } \right)\left( {\begin{array}{*{20}c} {K_{D}^{ - } - x + n_{ - , + } } \\ {n_{ - , - } } \\ \end{array} } \right)\left( {\begin{array}{*{20}c} {K_{D}^{0} - n_{ - ,0} } \\ {n_{ - ,0} } \\ \end{array} } \right)}}{{\left( {\begin{array}{*{20}c} {n - K_{H}^{ + } } \\ {K_{H}^{ - } } \\ \end{array} } \right)}}$$

where $Q = \{ K_{D}^{ + } ,K_{D}^{ - } ,n_{ + , + } ,n_{ - , - } ,n_{ - , + }$}, such that $x \ge \hat{x}$. Therefore, at difference with Eq. (9), quantities $K_{D}^{ + }$ and $K_{D}^{ - }$ can vary, and the sum is carried over all possible values of parameters such that $x \ge \hat{x}$, under the constrain $K_{D}^{ + } + K_{D}^{ - } + K_{D}^{0} = n$. In this manuscript, the Hy-test refers to Eq. (10). We use this test on a large set of genes, therefore a multiple comparison correction is required. In all subsequent analysis statistical significance indicates that a Bonferroni corrected p-value is below the 5% level^13^.

### Preprocessing procedure for microarray data

To test the effectiveness of the proposed method, we consider gene expression profiles of breast cancer (BRCA) cells in a pattern of paired tissues; 17.632 genes have been recorded in 75 tumour tissues and in the 75 paired normal tissues. Then the analysis has also been performed by considering 67 kidneys with renal clear cell carcinoma—KIRC—paired with 67 normal tissues. Data has been downloaded from The Cancer Genome Atlas (TCGA) database﻿ using the TCGA-assembler tool^14^. The expression profiles of duplicated genes have been replaced by their mean expression. Moreover, the expression of each gene has been normalized using a quantile normalization procedure implemented in R package preprocessCore^15^. Finally, gene expression values were log2-transformed.

### Quantitative analysis of GO-terms

The performance of the Hy-test has been compared to ﻿one of two classical methods of differential expression analysis, i.e., moderated t-test^4^ in combination with fold change larger than 2 and significance analysis of microarray^5^. Both tests are available from the Bioconductor repository﻿ and are implemented in the packages “limma” and “siggenes”, respectively. According to the three methods, genes that turned out to be significant were also compared by exploiting their functional roles with a Gene Ontology (GO) enrichment analysis^16^. We obtained three separate lists of significant GO-terms from the three sets of differentially expressed genes. GO-analysis has been done using the topGO package from Bioconductor, focusing on biological process terms. Fisher exact *p*-values have been associated with each GO-term. To identify GO-terms (e.g., cell cycle) conceptually associated with a specific cell line (for example, breast cancer), we have defined a novel procedure that counts the PubMed articles related to the biological concepts under exam, for example, breast cancer and cell cycle. We assume that more articles related to both concepts indicate a stronger conceptual association between them. The automated PubMed search has been carried out using the R package RISmed^17^. The used query considers articles published between January 2000 and December 2020. The probability of observing $n_{C,T}$ PubMed articles with both keywords “breast cancer” and “cell cycle” is

12 $$\Pr \left( {N_{C,T} = n_{C,T} {|}N,N_{C} ,N_{T} } \right) = \frac{{\left( {\begin{array}{*{20}c} {N_{C} } \\ {n_{C,T} } \\ \end{array} } \right)\left( {\begin{array}{*{20}c} {N - N_{C} } \\ {N_{T} - n_{C,T} } \\ \end{array} } \right)}}{{\left( {\begin{array}{*{20}c} N \\ {N_{T} } \\ \end{array} } \right)}}$$

where $N$ is the number of articles available on PubMed, $N_{C}$ is the number of articles with the keyword “breast cancer” and $N_{T}$ is the number of articles with “cell cycle” as keywords. Using a hypergeometric test we have associated a p-value of conceptual association with each GO term as

13 $$\Pr \left( {N_{C,T} \ge n_{C,T} } \right) = \mathop \sum \limits_{{X = n_{C,T} }}^{{{\text{min}}\left( {NC,NT} \right)}} \Pr \left( {X{|}N,N_{C} ,N_{T} } \right).$$

## Results

**Figure 1:**
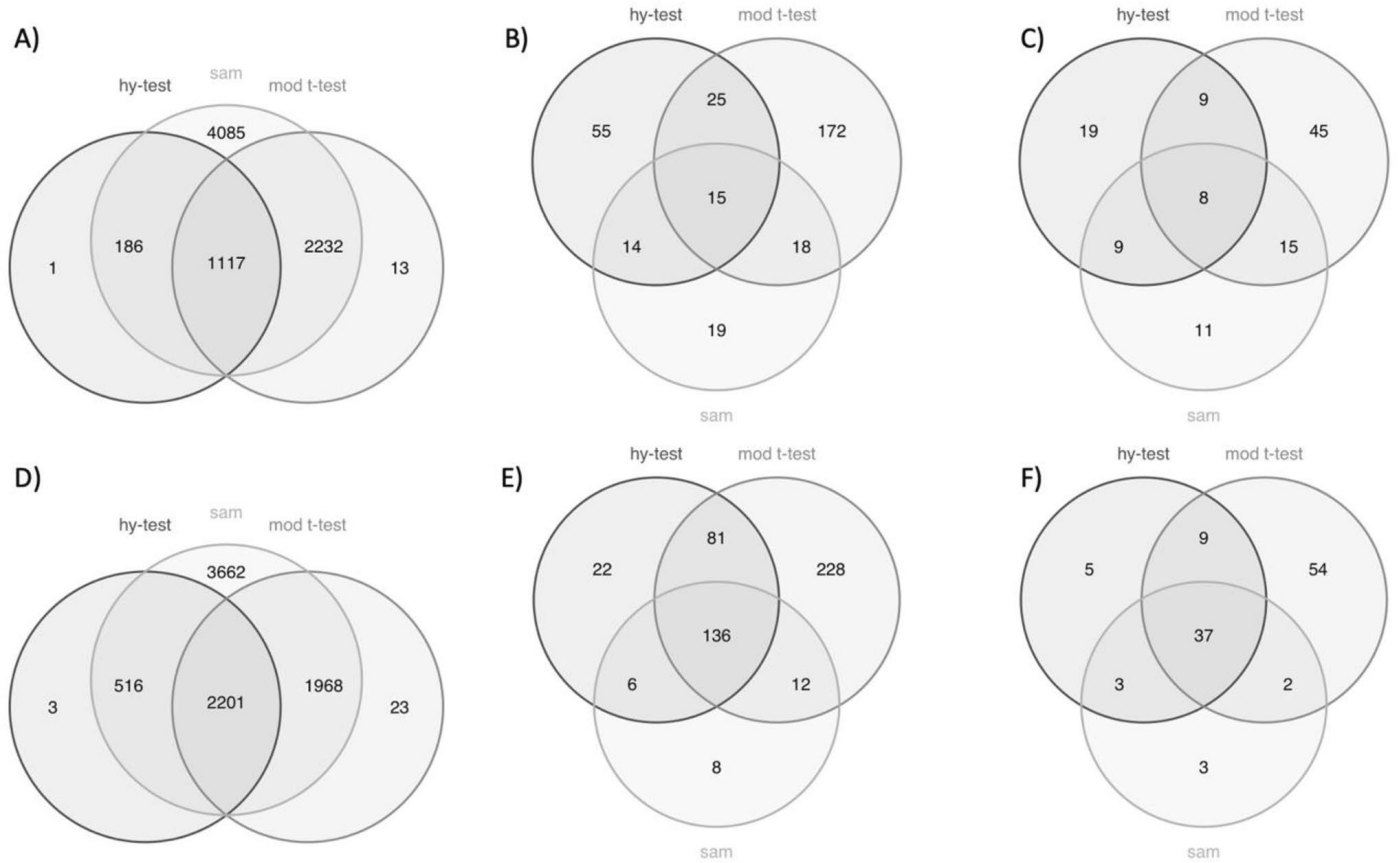
Venn diagrams of the differentially expressed genes and significant terms found in each of the three analysis steps by the three methods: Hy-test, moderated t-test, and SAM. The upper panels (**A**, **B**, **C**) refer to the breast tissue and the lower panels (**D**, **E**, **F**) to the kidney. The first column (**A** and **D**) refers to the DE analysis, the second column (**B** and **E**) to the enrichment analysis and the third column (**C** and **F**) to the PubMed research. Significance is assessed when a Bonferroni corrected p-value is below the 5% level.

The three methods, i.e. Hy-test, moderated t-test and SAM, have been compared. Venn diagrams reported in Fig. 1 clearly show the differences between the outcomes of the three considered methods.

Considering breast (kidney) tissues, the Hy-test identifies 1.304 (2.720) significant genes, whereas both SAM and the moderated t-test select many more genes: 7.620 (8.347)﻿ and 3.362 (4.192) significant genes, respectively. More importantly, panels A (breast cancer) and D (kidney cancer) of Fig. 1 clearly show that the Hy-test mostly identifies differentially expressed genes also identified by both the other methods. These results indicate that the Hy-test is more conservative than the other two tests. According to a GO-enrichment analysis of the lists of differentially expressed genes, 109 (245) significant terms result from the Hy-test gene list, 230 (457) from the moderated t-test list, and 66 (162) from the SAM list. The intersections among the detected lists of terms are pictured in Fig. 1B, E. A selection of significant terms with breast (kidney) cancer evaluated by researching PubMed papers﻿ is reported in Table 1 (Table 2).

**Table 1:** GO-terms significantly associated with breast cancer among significant GO-terms found using Hy-test, moderated t-test and both procedures.

Sign. GO-term	GO ID	Analysis	Term size	BR term size	*p* value
Cell cycle checkpoint signaling	GO:0000075	*Hy*-test	167	34	< 1.11E−16
Mitotic spindle checkpoint signaling	GO:0071174	*Hy*-test	38	14	< 1.11E−16
Regulation of cell cycle	GO:0051726	*Hy*-test	951	134	< 1.11E−16
Regulation of cell cycle process	GO:0010564	*Hy*-test	594	102	< 1.11E−16
Spindle assembly checkpoint signaling	GO:0071173	*Hy*-test	37	15	< 1.11E−16
Cell surface receptor signaling pathway	GO:0007166	Mod *t*-test	2485	643	< 1.11E−16
Cell–cell signaling	GO:0007267	Mod *t*-test	1545	436	< 1.11E−16
Regulation of signal transduction	GO:0009966	Mod *t*-test	2734	619	< 1.11E−16
Regulation of signaling	GO:0023051	Mod *t*-test	3107	719	< 1.11E−16
Signal transduction	GO:0007165	Mod *t*-test	5175	1210	< 1.11E−16
Angiogenesis	GO:0001525	Both	493	171	< 1.11E−16
Cell communication	GO:0007154	Both	5681	1342	< 1.11E−16
Cell population proliferation	GO:0008283	Both	1835	473	< 1.11E−16
Mitotic cell cycle	GO:0000278	Both	833	217	< 1.11E−16
Tissue development	GO:0009888	Both	1749	483	< 1.11E−16

Term size is the number of genes that compose a GO-term; BR term size is the number of GO-term genes associated with breast cancer; p-value is computed by using the hypergeometric distribution.

**Table 2:** GO-terms significantly associated with “kidney cancer” among significant GO-terms found using Hy-test, *t*-test and both procedures.

Sign. GO-term	GO ID	Analysis	Term size	KIRC term size	*p* value
Apoptotic process	GO:0006915	*Hy*-test	1761	363	< 1.11E−16
Cell death	GO:0008219	*Hy*-test	1951	396	< 1.11E−16
Programmed cell death	GO:0012501	*Hy*-test	1808	371	< 1.11E−16
Cell differentiation	GO:0030154	Mod *t*-test	3844	1159	< 1.11E−16
Kidney development	GO:0001822	Mod *t*-test	283	115	< 1.11E−16
Kidney epithelium development	GO:0072073	Mod t-test	133	61	< 1.11E−16
Regulation of cell differentiation	GO:0045595	Mod *t*-test	1432	459	1.98E−05
Renal system development	GO:0072001	Mod t-test	292	118	< 1.11E−16
Antigen processing and presentation	GO:0019882	Both	102	54	2.37E−09
Cell killing	GO:0001906	Both	173	79	6.80E−15
Immune system development	GO:0002520	Both	881	301	6.86E−04
Leukocyte mediated cytotoxicity	GO:0001909	Both	117	62	7.01E−09
Lymphocyte proliferation	GO:0046651	Both	276	133	4.07E−07
Regulation of signaling	GO:0023051	Both	3110	924	< 1.11E−16

Term size is the number of genes that compose a GO-term; KIRC term size is the number of GO-term genes associated with kidney cancer; *p*-value is computed by using the hypergeometric distribution.

The list of all terms is reported in Supplementary Table S1 (Table S2). Just 8 (37) of those terms have been found by all the methods, as shown in Fig. 1C﻿, F. It’s worth noticing that SAM analysis provides such a large number of differentially expressed genes, more than 5000 in both the applications, that it is reasonable to assume the presence of many false positives, while the Hy-test alone﻿ or the combined use of Hy-test and moderate t-test suggest better recovery of significant terms associated with both types of cancer.

A crucial issue in interpreting results from transcriptomics studies is the bias due to the significantly high and increasing number of cancer-related studies﻿ with respect to any other disease^18^. The consequence is that almost any gene has been (or will be) associated with cancer. Evaluating the performance of our algorithm by measuring its ability to retrieve cancer-related genes might not be sufficient. On the other hand, several different perturbations can trigger concerted “expression waves” marking state transitions that could cause global transcriptomic changes with common underlying characteristics^19^. The consequence, in this case, is the reported presence of a “generic signature” of differentially expressed genes, i.e. genes that are frequently detected as differentially expressed, despite the comparison performed^20^. Therefore, we evaluated the algorithms by considering their ability to avoid the selection of the generic signature, not because the genes selected are not related to the comparisons we are performing, but by testing which algorithm can retrieve more specific features of the system under investigation and not the effect of the generic perturbation. To measure the condition specificity of the used tests, i.e., the ability to select differentially expressed genes specifically related to the performed sample’s comparisons, we used the DE prior score defined and computed in^20^. The genes selected with the SAM test show a DE prior score cumulative distribution very close to the diagonal, explained by selecting a high number of genes, most of which are probably false positive (Supplementary Fig. 1). The DE prior scores of the genes selected as differentially expressed in breast cancer tissues with the Hy-test and the moderated t-test are similarly distributed. On the other hand, the Hy-test in kidney data analysis selects differentially expressed genes with significantly lower DE prior scores. Even though the Hy-test selects a smaller number of differentially expressed genes, its focus is not on the genes that appear differentially expressed in any condition of comparison but, at least in these examples, on genes more peculiar to the system under investigation.

### Correlation structures and spectral analysis

**Figure 2:**
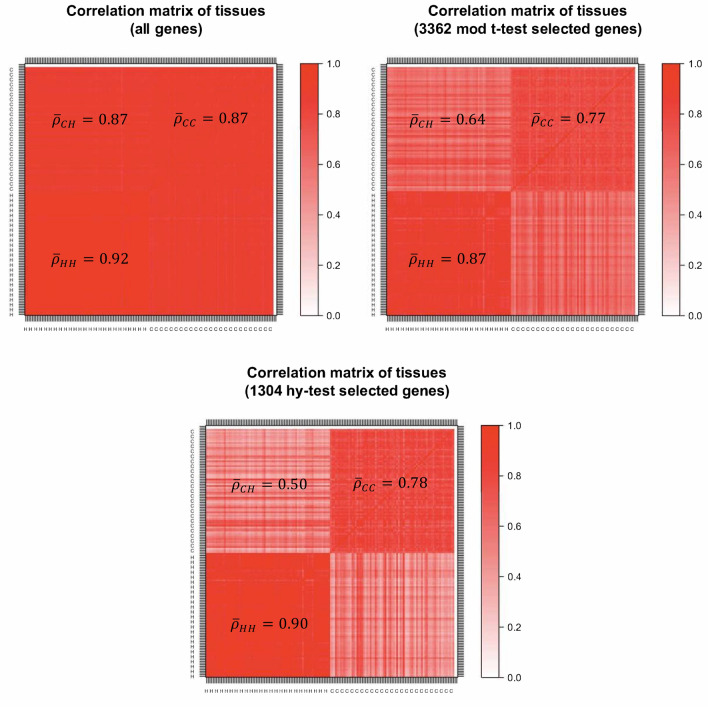
Correlation structure of breast cancer expression genes. Top-left panel refers to all genes, the top-right panel refers to the set of genes selected by moderated t-test, and the bottom panel refers to the set of genes selected by the Hy-test. ${\bar{\varrho}}$ is the block average correlation.

**Figure 3:**
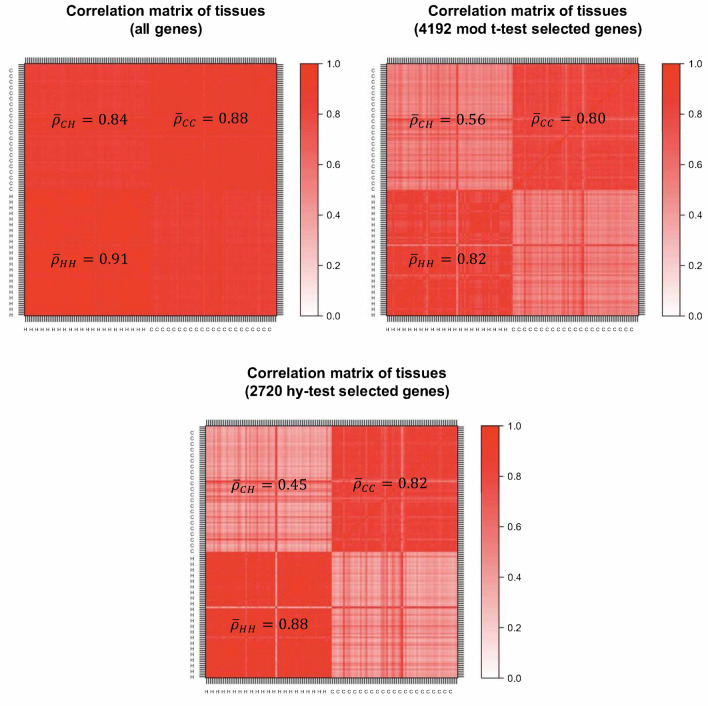
Correlation structure of kidney cancer expression genes. Top-left panel refers to all genes, the top-right panel refers to the set of genes selected by moderated t-test, and the bottom panel refers to the set of genes selected by the Hy-test. ${\bar{\varrho}}$ is the block average correlation.

Besides using statistical techniques to identify differentially expressed genes, it is also important to use statistical charts to detect normalization problems, differential expression designation problems, and common analysis errors. For example, as shown in Fig. 2 (Fig. 3) for breast (kidney)-cancer data, a simple comparison between the correlation matrices of tissues is﻿ constructed by using (1) all available genes, (2) the genes selected by the moderate t-test and (3) the ones selected by the Hy-test, allows one to perform a quality check on the two analyses of differential expression. Specifically, it is possible to observe how the panel of genes selected by the Hy-test can be considered a better filter than the one obtained through the moderated t-test since the former amplifies gene expression differences between the two types of tissues-healthy (H) and cancer (C) tissues. Furthermore, results reported in Fig. 3 about kidney cancer data suggest a misclassification of one (H, C)-pair tissue, namely, TCGA.CW.5591, which corresponds to the straight lines of opposite colours in the figure.

Many times genes do not work in isolation, but their “effect” is organised into “eigengene” modes, which one can study by performing a Principal Component Analysis (PCA)^21^. The first component typically reflects the batch effect corresponding to the “average expression profile” of genes, whereas minor components may identify disease (or any other perturbation) effects^22^. The dimensionality reduction obtained by considering principal components provides relevant insights into the considered selection procedures of differentially expressed genes. Using all the genes, the first eigenvector, which explains about 90% of the total variance, also captures the differential effect of the two types of paired tissues as a background effect, making it impossible to use it to identify the gene-disease association. However, analysing the two reduced sets of genes that we identified through the moderated t-test and the Hy-test, we observe that the two effects (background and difference between healthy and cancer tissues) are split into the first two principal components. The variance explained by the two components together is the same as the one explained by the first component obtained from the whole dataset, i.e., about 90%. When looking at the distribution of gene scores projected on the first component (top panels of Supplementary Figs. 2a and 3a), we note a peak in the right tail of the distribution, which smooths out if one considers only genes selected through the t-test, and eventually disappears if one only focuses on genes selected through the Hy-test (batch effect). This evidence suggests ﻿using the second principal component (bottom panels of Supplementary Figs. 2a and 3a) to obtain more insights into the involvement of the selected genes in the differentiation between the two types of tissue. Remarkably, the second component for the Hy-test selected genes explains about twice the variance that the same component for t-test selected genes does. In the Supplementary Material, we report the first two eigenvectors (Supplementary Figs. 2a and 3a) and the correlation structures obtained by ordering the genes according to the second principal component scores (Supplementary Figs. 2b and 3b). This unsupervised procedure can be used in conjunction with ours to visualise high-dimensional space better and investigate the structure of several complex systems in biology^23^.

### Robustness analysis

To evaluate the finite sample properties of our test, we perform a Monte Carlo simulation in various scenarios. We assessed the performance of both the Hy-test and moderated t-test in terms of power functions (i.e., rejection rate) under the null (a) and alternative (b) hypotheses, respectively. Simulations are performed by generating paired vectors $\left( {Y_{1} , Y_{2} } \right)$ of length $n \in \left\{ {50, 75} \right\}$ of synthetic expression profiles, once log-normally distributed and once power-law distributed. ($Y_{1} \sim PL\left( {x_{{{\text{min}}}} = 20, \alpha = 3.5} \right)$ and $Y_{2} \sim PL\left( {x_{{{\text{min}}}} = 40, \alpha = 3.5} \right)$) Under the null hypothesis we simulated two independent samples, {$Y_{1}$} and {$Y_{2}$}, such that (1) $E\left[ {Y_{2} } \right] - E\left[ {Y_{1} } \right] = 0$ and (2) $Var\left[ {Y_{1} } \right] = Var\left[ {Y_{2} } \right] = 0.25$, whereas under the alternative we considered (1) $E\left[ {Y_{2} } \right] - E\left[ {Y_{1} } \right] = 1$ and (2) $Var\left[ {Y_{1} } \right] = Var\left[ {Y_{2} } \right] = 0.25$. For the latter, we assessed the sensitivity of both methods, the Hy-test and moderated t-test, according to three different correlation structures among the synthetic paired tissues, i.e., $\rho \in \left\{ {0.1, 0.2, 0.4} \right\}$. For each block of simulations, we performed 250 Monte Carlo replicates. Table 3 shows the mean rejection rate after adjusting the *p*-values with the Bonferroni correction. Results of simulations under the null hypothesis of no differential expression block (a) of simulations are not shown because both tests were robust in detecting true negatives.

**Table 3:** Results of simulation block (b), where two vectors of paired synthetic expression profiles $\left( {{\text{Y}}_{1} ,{\text{ Y}}_{2} } \right)$, have to satisfy (1) ${\text{E}}\left[ {{\text{Y}}_{2} } \right] - {\text{E}}\left[ {{\text{Y}}_{1} } \right] = 1$ and (2) ${\text{Var}}\left[ {{\text{Y}}_{1} } \right] = {\text{Var}}\left[ {{\text{Y}}_{2} } \right] = 0.25$.

	$Cor\left( {Y_{1} , Y_{2} } \right) = 0.1$		$Cor\left( {Y_{1} , Y_{2} } \right) = 0.2$		$Cor\left( {Y_{1} , Y_{2} } \right) = 0.4$	
	Log-normal	Power-law	Log-normal	Power-law	Log-normal	Power-law
**n = 50**						
Hy-test	0.89	0.94	0.94	0.97	1.00	0.99
Mod t-test	0.85	0.70	0.96	0.88	1.00	0.98
**n = 75**						
Hy-test	1.00	1.00	1.00	1.00	1.00	1.00
Mod t-test	0.90	0.76	0.98	0.93	1.00	1.00

Average rejection rates after 250 Monte Carlo replicates is reported for two different sample sizes, i.e., ${\text{n}} \in \left\{ {50, \,75} \right\}$, and distributions (log-normal and power-law), after adjusting the p-values with the Bonferroni correction.

According to the results reported in Table 3, the Hy-test method shows ﻿greater robustness than the moderate t-test in identifying true positives, even in low correlation and especially with highly leptokurtic distributions, such as the power-law distribution.

## Discussion

DEA plays a central role in comparative transcriptomic studies, which represent the vast majority of gene expression analyses. The core action that defines a transcriptomic comparative study is the definition and retrieval of differentially expressed genes in different conditions. Working with data generated by a plethora of procedures in a very noisy and variable system, such as a biological one, requires one to adopt different approaches to analyse a given phenomenon. We provide a biological interpretation of the results obtained by performing a differential expression analysis of breast and kidney cancer genes through the moderated t-test and our Hy-test.

In the case of the real breast cancer profiles analysed, both moderated t-test and Hy-test reveal that DE genes are enriched in functions involved in tissue development and cell proliferation, as expected^24^. While the t-test approach focuses on signal transduction^25–27^, the Hy-test highlights a central role in regulating the cell cycle in breast cancer, as strongly supported by recent literature^28,29^.

In detail, the mammary gland is a tissue characterised by a high proliferation rate, and the developmental programs are prompt to be subverted to promote cancer progression. In the gland, many cells are extremely polarised. When extrinsic or intrinsic factors disrupt the maintenance of this organisation, this disruption may act as a promoter of hyperplasia and transformation^30^. Several studies also suggest that the disruption of the typical apical-basal polarity may contribute to the metastatic event^31^. The deregulation of extracellular matrix proteins and signalling is sufficient to promote breast cancer development and progression^24^. Signal transduction has a central role in breast cancer; indeed, breast cancer molecular classification usually follows the presence or absence of specific hormone and growth factor receptors^25,26^ with direct implications in diagnosis, prognosis, and therapy. Both tissue development and signal transduction have a central role in breast cancer. However, the moderated t-test is not efficient in retrieving the cell-intrinsic cell cycle deregulation GO terms that the Hy-test has pinpointed. Indeed, cell cycle deregulation is crucial to breast cancer development and cell cycle control machinery targets novel therapeutic strategies, such as CDK4/6 inhibitors^28,29^.

In the case of kidney cancers, the differences between the Hy-test results and those from the moderated t-test are even more apparent. Both approaches retrieve an enrichment in cell signalling, particularly in the contest of the immunological microenvironment^32,33^, and the t-test only finds the involvement of functions related to kidney development^34^. However, Hy-test only points to “programmed cell death” , which is a central mechanism in kidney cancer, targeted by some therapeutic approaches to the disease^33^.

In detail, it is known that the reshaping of the metabolism is one of the key steps that kidney tumour cells must undergo during cancer progression. This metabolic reshape strongly relies on the cross-talk between cancer cells and the tumour microenvironment^35^. In particular, the inflammatory microenvironment is involved in developing of both pre-neoplastic alterations and kidney cancer^36^. To further support our findings, we can also mention that, for patients with renal clear cell carcinoma, a model has been proposed based on a few immune-related genes that can predict the prognosis based on tumour immune microenvironments^37^. Considering that the programmed cell death subversion plays a central role in kidney cancer development, it is intriguing to ascertain that only the Hy-test leads to retrieving this GO term from the enrichment analysis, strongly suggesting that a dual approach using both the Hy-test and moderated t-test can be even more suitable than single methods alone to investigate the biological meaning of a DEA on real data.

## Conclusions

Hy-test can be adopted alone or jointly with other existing DEA tests to identify differentially expressed genes in a very conservative way, as confirmed by the analyses of real data of breast and kidney cancers reported in this paper. Such robust information would remain otherwise hidden within the extremely large number of genes identified by standard DEA tests as differentially expressed, likely including many false positives. According to our results, the moderated t-test increases substantially the number of significant genes retrieved from DEA with respect to the Hy-test, broadening the differential gene ontology enrichment. Consequently, the Hy-test is more selective than moderated t-test in both retrieving DE genes and relevant terms of GO. On the other end, the SAM test detects even more statistically significant genes than the moderated t-test, leading to apparent issues in identifying of enriched GO terms. To evaluate the performance of the analysed DEA tests in detecting cancer-related genes, we have focused on the enriched ontology terms validated through the automated PubMed-search procedure described in the “Methods”section. In this way, we can focus our attention only on terms with a widely established involvement in cancer diseases. The excluded terms might also carry important cancer information, but their analysis goes beyond the purpose of the present performance evaluation. Hy-test is not only able to narrow the window of selected genes but focusing the functional analysis. It can also retrieve specific terms of GO that would be otherwise missing. This is particularly evident in the breast cancer dataset, where the moderated t-test also collects the vast majority of DE genes retrieved by the Hy-test. However, the enrichment analysis shows only a moderate overlapping, strongly suggesting that Hy-test can retrieve a different set of genes that points to functions of biological relevance that would be otherwise missed. This is also true to a lower extent for the kidney dataset.

## Supplementary Information

Supplementary Information.

## Data Availability

Our source codes and data are available for downloading in the GitHub repository (https://github.com/gianluca-sottile/A-Novel-Statistical-Test-For-Differential-Expression-Analysis).

## References

[1] Cui X, Churchill GA (2003). Statistical tests for differential expression in cDNA microarray experiments. Genome Biol..

[2] Pan W (2002). A comparative review of statistical methods for discovering differentially expressed genes in replicated microarray experiments. Bioinformatics.

[3] Fagerland MW, Sandvik L (2009). Performance of five two-sample location tests for skewed distributions with unequal variances. Contemp. Clin. Trials.

[4] Smyth GK (2004). Linear models and empirical bayes methods for assessing differential expression in microarray experiments. Stat. Appl. Genet. Mol. Biol..

[5] Tusher VG, Tibshirani R, Chu G (2001). Significance analysis of microarrays applied to the ionizing radiation response. Proc. Natl. Acad. Sci..

[6] Gallo CA, Cecchini RL, Carballido JA, Micheletto S, Ponzoni I (2016). Discretization of gene expression data revised. Brief. Bioinform..

[7] Dussaut, J. S., Gallo, C. A., Carballido, J. A. & Ponzoni, I. Analysis of Gene Expression Discretization Techniques in Microarray Biclustering. in *International Conference on Bioinformatics and Biomedical Engineering* 257–266 (Springer, 2017).

[8] Karlebach G, Shamir R (2008). Modelling and analysis of gene regulatory networks. Nat. Rev. Mol. cell Biol..

[9] Dimitrova ES, Licona MPV, McGee J, Laubenbacher R (2010). Discretization of time series data. J. Comput. Biol..

[10] McCarthy DJ, Smyth GK (2009). Testing significance relative to a fold-change threshold is a TREAT. Bioinformatics.

[11] Catlett J (1991). On Changing Continuous Attributes Into Ordered Discrete Attributes. European Working Session on Learning.

[12] Whitley D (1994). A genetic algorithm tutorial. Stat. Comput..

[13] Miller RG (1981). Simultaneous Statistical Inference.

[14] Wei L (2018). TCGA-assembler 2: Software pipeline for retrieval and processing of TCGA/CPTAC data. Bioinformatics.

[15] Bolstad BM, Irizarry RA, Åstrand M, Speed TP (2003). A comparison of normalization methods for high density oligonucleotide array data based on variance and bias. Bioinformatics.

[16] Zheng Q, Wang X-J (2008). GOEAST: A web-based software toolkit for Gene Ontology enrichment analysis. Nucleic Acids Res..

[17] Kovalchik, S. RISmed: Download Content from NCBI Databases. *R package version 2.3.0* https://cran.r-project.org/package=RISmed (2021).

[18] de Magalhães, J. P. Every gene can (and possibly will) be associated with cancer. *Trends Genet.* (2021). 10.1016/j.tig.2021.09.005 34756472

[19] Zimatore G, Tsuchiya M, Hashimoto M, Kasperski A, Giuliani A (2021). Self-organization of whole-gene expression through coordinated chromatin structural transition. Biophys. Rev..

[20] Crow M, Lim N, Ballouz S, Pavlidis P, Gillis J (2019). Predictability of human differential gene expression. Proc. Natl. Acad. Sci..

[21] Roden JC (2006). Mining gene expression data by interpreting principal components. BMC Bioinform..

[22] Censi F, Calcagnini G, Bartolini P, Giuliani A (2010). A systems biology strategy on differential gene expression data discloses some biological features of atrial fibrillation. PLoS ONE.

[23] Langfelder P, Horvath S (2007). Eigengene networks for studying the relationships between co-expression modules. BMC Syst. Biol..

[24] Zhu J, Xiong G, Trinkle C, Xu R (2014). Integrated extracellular matrix signaling in mammary gland development and breast cancer progression. Histol. Histopathol..

[25] Akram M, Iqbal M, Daniyal M, Khan AU (2017). Awareness and current knowledge of breast cancer. Biol. Res..

[26] Tan, P. H. *et al.* The 2019 World Health Organization classification of tumours of the breast. (2020). 10.1111/his.14091 32056259

[27] Rajan A (2021). Deregulated estrogen receptor signaling and DNA damage response in breast tumorigenesis. Biochim. Biophys. Acta (BBA) Rev. Cancer.

[28] Thu KL, Soria-Bretones I, Mak TW, Cescon DW (2018). Targeting the cell cycle in breast cancer: Towards the next phase. Cell Cycle.

[29] Ding L (2020). The roles of cyclin-dependent kinases in cell-cycle progression and therapeutic strategies in human breast cancer. Int. J. Mol. Sci..

[30] Rejon C, Al-Masri M, McCaffrey L (2016). Cell polarity proteins in breast cancer progression. J. Cell. Biochem..

[31] Chatterjee SJ, McCaffrey L (2014). Emerging role of cell polarity proteins in breast cancer progression and metastasis. Breast Cancer Targets Ther..

[32] Drake CG, Stein MN (2018). The immunobiology of kidney cancer. J. Clin. Oncol..

[33] Aggen DH, Drake CG, Rini BI (2020). Targeting PD-1 or PD-L1 in metastatic kidney cancer: Combination therapy in the first-line setting. Clin. Cancer Res..

[34] Drake KA (2020). Stromal β-catenin activation impacts nephron progenitor differentiation in the developing kidney and may contribute to Wilms tumor. Development.

[35] Wettersten HI (2020). Reprogramming of metabolism in kidney cancer. Semin. Nephrol..

[36] Peterfi L, Yusenko MV, Kovacs G (2019). IL6 shapes an inflammatory microenvironment and triggers the development of unique types of cancer in end-stage kidney. Anticancer Res..

[37] Zou Y, Hu C (2020). A 14 immune-related gene signature predicts clinical outcomes of kidney renal clear cell carcinoma. PeerJ.

