# Supplementary material for: A multivariate statistical test for differential expression analysis
Source: Sci Rep. 2022 May 18;12:8265. doi: 10.1038/s41598-022-12246-w (PMC9117296; doi:10.1038/s41598-022-12246-w)
Supplement: Supplementary file 1 — Supplementary Information. [file 41598_2022_12246_MOESM1_ESM.docx]

# Supplementary Information

Supplementary Table S1. GO-terms significantly associated with breast cancer among significant GO-terms found using Hy-test, moderated t-test and both procedures.. Term size is the number of genes that compose a GO-term; BR term size is the number of GO-term genes associated with breast cancer.

| Sign. GO-term | GO_ID | Analysis | term size | BR term size | p-value |
| --- | --- | --- | --- | --- | --- |
| cell cycle | GO:0007049 | hy-test | 1625 | 224 | <1.11E-16 |
| cell cycle checkpoint signaling | GO:0000075 | hy-test | 167 | 32 | <1.11E-16 |
| cell cycle G2/M phase transition | GO:0044839 | hy-test | 142 | 28 | <1.11E-16 |
| cell cycle phase transition | GO:0044770 | hy-test | 472 | 77 | <1.11E-16 |
| cell cycle process | GO:0022402 | hy-test | 1092 | 177 | <1.11E-16 |
| cellular component organization or biogenesis | GO:0071840 | hy-test | 5705 | 523 | <1.11E-16 |
| chromosome organization | GO:0051276 | hy-test | 776 | 112 | 8.01E-06 |
| DNA replication | GO:0006260 | hy-test | 254 | 43 | <1.11E-16 |
| growth | GO:0040007 | hy-test | 881 | 105 | <1.11E-16 |
| mitotic cell cycle checkpoint signaling | GO:0007093 | hy-test | 127 | 26 | <1.11E-16 |
| mitotic cell cycle phase transition | GO:0044772 | hy-test | 385 | 67 | 1.57E-03 |
| mitotic spindle checkpoint signaling | GO:0071174 | hy-test | 38 | 14 | <1.11E-16 |
| negative regulation of cell cycle | GO:0045786 | hy-test | 348 | 58 | <1.11E-16 |
| negative regulation of cell cycle phase transition | GO:1901988 | hy-test | 223 | 42 | <1.11E-16 |
| negative regulation of cell cycle process | GO:0010948 | hy-test | 263 | 50 | <1.11E-16 |
| negative regulation of chromosome segregation | GO:0051985 | hy-test | 42 | 15 | 2.09E-02 |
| negative regulation of mitotic cell cycle | GO:0045930 | hy-test | 211 | 40 | <1.11E-16 |
| positive regulation of cell cycle | GO:0045787 | hy-test | 286 | 46 | <1.11E-16 |
| positive regulation of cell cycle process | GO:0090068 | hy-test | 212 | 39 | <1.11E-16 |
| protein phosphorylation | GO:0006468 | hy-test | 1479 | 160 | <1.11E-16 |
| regulation of cell cycle | GO:0051726 | hy-test | 951 | 134 | <1.11E-16 |
| regulation of cell cycle phase transition | GO:1901987 | hy-test | 346 | 59 | <1.11E-16 |
| regulation of cell cycle process | GO:0010564 | hy-test | 594 | 102 | <1.11E-16 |
| regulation of chromosome organization | GO:0033044 | hy-test | 179 | 33 | <1.11E-16 |
| regulation of mitotic cell cycle | GO:0007346 | hy-test | 415 | 70 | <1.11E-16 |
| regulation of mitotic nuclear division | GO:0007088 | hy-test | 108 | 27 | <1.11E-16 |
| regulation of nuclear division | GO:0051783 | hy-test | 133 | 29 | <1.11E-16 |
| spindle assembly checkpoint signaling | GO:0071173 | hy-test | 38 | 14 | <1.11E-16 |
| biological adhesion | GO:0022610 | mod t-test | 1407 | 409 | <1.11E-16 |
| biological regulation | GO:0065007 | mod t-test | 10967 | 2280 | <1.11E-16 |
| cell adhesion | GO:0007155 | mod t-test | 1400 | 405 | <1.11E-16 |
| cell development | GO:0048468 | mod t-test | 1983 | 485 | <1.11E-16 |
| cell differentiation | GO:0030154 | mod t-test | 3827 | 910 | <1.11E-16 |
| cell migration | GO:0016477 | mod t-test | 1373 | 377 | <1.11E-16 |
| cell motility | GO:0048870 | mod t-test | 1542 | 415 | <1.11E-16 |
| cell surface receptor signaling pathway | GO:0007166 | mod t-test | 2485 | 643 | <1.11E-16 |
| cell surface receptor signaling pathway involved in cell-cell signaling | GO:1905114 | mod t-test | 519 | 144 | 5.41E-03 |
| cell-cell adhesion | GO:0098609 | mod t-test | 831 | 237 | <1.11E-16 |
| cell-cell signaling | GO:0007267 | mod t-test | 1545 | 436 | <1.11E-16 |
| cellular hormone metabolic process | GO:0034754 | mod t-test | 128 | 53 | <1.11E-16 |
| cellular response to nitrogen compound | GO:1901699 | mod t-test | 651 | 177 | 7.03E-03 |
| epithelial cell differentiation | GO:0030855 | mod t-test | 590 | 161 | <1.11E-16 |
| epithelium development | GO:0060429 | mod t-test | 1035 | 264 | 7.27E-13 |
| extracellular matrix organization | GO:0030198 | mod t-test | 284 | 123 | 1.79E-10 |
| G protein-coupled receptor signaling pathway | GO:0007186 | mod t-test | 892 | 237 | <1.11E-16 |
| hormone metabolic process | GO:0042445 | mod t-test | 207 | 72 | <1.11E-16 |
| localization of cell | GO:0051674 | mod t-test | 1542 | 415 | <1.11E-16 |
| lung development | GO:0030324 | mod t-test | 172 | 59 | <1.11E-16 |
| multicellular organismal process | GO:0032501 | mod t-test | 6713 | 1599 | 1.56E-10 |
| negative regulation of angiogenesis | GO:0016525 | mod t-test | 98 | 40 | <1.11E-16 |
| negative regulation of cell population proliferation | GO:0008285 | mod t-test | 683 | 185 | <1.11E-16 |
| negative regulation of cellular process | GO:0048523 | mod t-test | 4510 | 985 | <1.11E-16 |
| negative regulation of vasculature development | GO:1901343 | mod t-test | 100 | 41 | 2.73E-04 |
| positive regulation of cell differentiation | GO:0045597 | mod t-test | 802 | 221 | <1.11E-16 |
| positive regulation of cell migration | GO:0030335 | mod t-test | 497 | 153 | <1.11E-16 |
| positive regulation of cell motility | GO:2000147 | mod t-test | 519 | 162 | <1.11E-16 |
| positive regulation of cell population proliferation | GO:0008284 | mod t-test | 880 | 252 | <1.11E-16 |
| positive regulation of cellular component movement | GO:0051272 | mod t-test | 531 | 168 | 2.36E-03 |
| positive regulation of cellular process | GO:0048522 | mod t-test | 5208 | 1122 | <1.11E-16 |
| positive regulation of epithelial cell proliferation | GO:0050679 | mod t-test | 184 | 62 | <1.11E-16 |
| positive regulation of locomotion | GO:0040017 | mod t-test | 533 | 166 | <1.11E-16 |
| positive regulation of phosphate metabolic process | GO:0045937 | mod t-test | 855 | 228 | 9.68E-05 |
| positive regulation of phosphorylation | GO:0042327 | mod t-test | 780 | 209 | <1.11E-16 |
| positive regulation of protein phosphorylation | GO:0001934 | mod t-test | 701 | 186 | <1.11E-16 |
| positive regulation of transferase activity | GO:0051347 | mod t-test | 537 | 147 | <1.11E-16 |
| regulation of angiogenesis | GO:0045765 | mod t-test | 271 | 91 | <1.11E-16 |
| regulation of cell communication | GO:0010646 | mod t-test | 3092 | 718 | <1.11E-16 |
| regulation of cell differentiation | GO:0045595 | mod t-test | 1428 | 377 | <1.11E-16 |
| regulation of cell migration | GO:0030334 | mod t-test | 856 | 245 | <1.11E-16 |
| regulation of cell motility | GO:2000145 | mod t-test | 910 | 263 | <1.11E-16 |
| regulation of cell population proliferation | GO:0042127 | mod t-test | 1570 | 425 | <1.11E-16 |
| regulation of cellular component movement | GO:0051270 | mod t-test | 981 | 281 | <1.11E-16 |
| regulation of cellular process | GO:0050794 | mod t-test | 9886 | 2067 | <1.11E-16 |
| regulation of epithelial cell proliferation | GO:0050678 | mod t-test | 326 | 98 | <1.11E-16 |
| regulation of hormone levels | GO:0010817 | mod t-test | 480 | 139 | <1.11E-16 |
| regulation of kinase activity | GO:0043549 | mod t-test | 724 | 196 | <1.11E-16 |
| regulation of localization | GO:0032879 | mod t-test | 2583 | 647 | <1.11E-16 |
| regulation of locomotion | GO:0040012 | mod t-test | 948 | 274 | <1.11E-16 |
| regulation of membrane potential | GO:0042391 | mod t-test | 410 | 128 | <1.11E-16 |
| regulation of signal transduction | GO:0009966 | mod t-test | 2734 | 619 | <1.11E-16 |
| regulation of signaling | GO:0023051 | mod t-test | 3107 | 719 | <1.11E-16 |
| regulation of vasculature development | GO:1901342 | mod t-test | 275 | 93 | 2.72E-05 |
| response to chemical | GO:0042221 | mod t-test | 3901 | 923 | <1.11E-16 |
| response to growth factor | GO:0070848 | mod t-test | 665 | 185 | <1.11E-16 |
| response to hormone | GO:0009725 | mod t-test | 831 | 218 | <1.11E-16 |
| response to ketone | GO:1901654 | mod t-test | 188 | 65 | 1.17E-16 |
| signal transduction | GO:0007165 | mod t-test | 5175 | 1210 | <1.11E-16 |
| transmembrane receptor protein tyrosine kinase signaling pathway | GO:0007169 | mod t-test | 587 | 166 | <1.11E-16 |
| angiogenesis | GO:0001525 | both | 493 | 171 | <1.11E-16 |
| cell communication | GO:0007154 | both | 5681 | 1342 | <1.11E-16 |
| cell division | GO:0051301 | both | 594 | 162 | <1.11E-16 |
| cell population proliferation | GO:0008283 | both | 1835 | 473 | <1.11E-16 |
| enzyme linked receptor protein signaling pathway | GO:0007167 | both | 900 | 259 | <1.11E-16 |
| locomotion | GO:0040011 | both | 1702 | 469 | <1.11E-16 |
| mitotic cell cycle | GO:0000278 | both | 833 | 217 | <1.11E-16 |
| mitotic cell cycle process | GO:1903047 | both | 681 | 181 | 1.72E-05 |
| mitotic nuclear division | GO:0140014 | both | 279 | 88 | <1.11E-16 |
| movement of cell or subcellular component | GO:0006928 | both | 1949 | 509 | <1.11E-16 |
| nuclear division | GO:0000280 | both | 421 | 126 | <1.11E-16 |
| regulation of phosphorylation | GO:0042325 | both | 1188 | 306 | <1.11E-16 |
| regulation of protein phosphorylation | GO:0001932 | both | 1046 | 270 | <1.11E-16 |
| regulation of transferase activity | GO:0051338 | both | 833 | 216 | <1.11E-16 |
| signaling | GO:0023052 | both | 5633 | 1337 | <1.11E-16 |
| tissue development | GO:0009888 | both | 1749 | 483 | <1.11E-16 |
| vasculature development | GO:0001944 | both | 689 | 234 | 1.05E-15 |

Supplementary Table S2. GO-terms significantly associated with “kidney cancer” among significant GO-terms found using Hy-test, moderated t-test and both procedures.

| Sign. GO-term | GO_ID | Analysis | term size | BR term size | p-value |
| --- | --- | --- | --- | --- | --- |
| apoptotic process | GO:0006915 | hy-test | 1761 | 363 | <1.11E-16 |
| cell death | GO:0008219 | hy-test | 1951 | 396 | <1.11E-16 |
| negative regulation of cellular process | GO:0048523 | hy-test | 4519 | 825 | <1.11E-16 |
| phosphorylation | GO:0016310 | hy-test | 1739 | 349 | <1.11E-16 |
| programmed cell death | GO:0012501 | hy-test | 1808 | 371 | <1.11E-16 |
| protein phosphorylation | GO:0006468 | hy-test | 1480 | 300 | <1.11E-16 |
| regulation of GTPase activity | GO:0043087 | hy-test | 328 | 87 | 1.94E-05 |
| small GTPase mediated signal transduction | GO:0007264 | hy-test | 489 | 123 | 4.08E-02 |
| amide transport | GO:0042886 | mod t-test | 289 | 105 | 2.20E-07 |
| angiogenesis | GO:0001525 | mod t-test | 493 | 179 | <1.11E-16 |
| anion transport | GO:0006820 | mod t-test | 488 | 170 | 5.46E-15 |
| antigen processing and presentation of peptide or polysaccharide antigen via MHC class II | GO:0002504 | mod t-test | 33 | 22 | <1.11E-16 |
| cell development | GO:0048468 | mod t-test | 1987 | 591 | <1.11E-16 |
| cell differentiation | GO:0030154 | mod t-test | 3844 | 1159 | <1.11E-16 |
| cell motility | GO:0048870 | mod t-test | 1543 | 525 | <1.11E-16 |
| cell-cell signaling | GO:0007267 | mod t-test | 1548 | 527 | 7.06E-08 |
| enzyme linked receptor protein signaling pathway | GO:0007167 | mod t-test | 900 | 276 | 5.90E-03 |
| epithelial cell proliferation | GO:0050673 | mod t-test | 382 | 136 | <1.11E-16 |
| epithelium development | GO:0060429 | mod t-test | 1041 | 325 | 6.68E-16 |
| G protein-coupled receptor signaling pathway | GO:0007186 | mod t-test | 910 | 301 | 4.00E-02 |
| gland development | GO:0048732 | mod t-test | 422 | 145 | <1.11E-16 |
| interleukin-2 production | GO:0032623 | mod t-test | 60 | 32 | 2.18E-05 |
| kidney development | GO:0001822 | mod t-test | 283 | 115 | <1.11E-16 |
| kidney epithelium development | GO:0072073 | mod t-test | 133 | 61 | <1.11E-16 |
| kidney morphogenesis | GO:0060993 | mod t-test | 91 | 43 | <1.11E-16 |
| localization of cell | GO:0051674 | mod t-test | 1543 | 525 | <1.11E-16 |
| locomotion | GO:0040011 | mod t-test | 1703 | 565 | <1.11E-16 |
| MAPK cascade | GO:0000165 | mod t-test | 728 | 240 | 4.04E-04 |
| mesonephros development | GO:0001823 | mod t-test | 95 | 44 | 9.38E-08 |
| movement of cell or subcellular component | GO:0006928 | mod t-test | 1950 | 613 | <1.11E-16 |
| nephron development | GO:0072006 | mod t-test | 137 | 66 | <1.11E-16 |
| nephron epithelium development | GO:0072009 | mod t-test | 106 | 49 | <1.11E-16 |
| nephron morphogenesis | GO:0072028 | mod t-test | 75 | 37 | <1.11E-16 |
| nephron tubule development | GO:0072080 | mod t-test | 89 | 42 | <1.11E-16 |
| nephron tubule morphogenesis | GO:0072078 | mod t-test | 70 | 35 | <1.11E-16 |
| organic acid transport | GO:0015849 | mod t-test | 296 | 106 | <1.11E-16 |
| positive regulation of cell migration | GO:0030335 | mod t-test | 497 | 187 | 2.64E-13 |
| positive regulation of cell motility | GO:2000147 | mod t-test | 519 | 198 | 1.26E-13 |
| positive regulation of gene expression | GO:0010628 | mod t-test | 1051 | 340 | <1.11E-16 |
| positive regulation of locomotion | GO:0040017 | mod t-test | 533 | 206 | 2.55E-11 |
| positive regulation of signal transduction | GO:0009967 | mod t-test | 1408 | 436 | 1.03E-03 |
| positive regulation of signaling | GO:0023056 | mod t-test | 1590 | 495 | 1.93E-05 |
| regulation of angiogenesis | GO:0045765 | mod t-test | 271 | 106 | <1.11E-16 |
| regulation of cell differentiation | GO:0045595 | mod t-test | 1432 | 459 | 1.98E-05 |
| regulation of cell migration | GO:0030334 | mod t-test | 856 | 298 | <1.11E-16 |
| regulation of cell motility | GO:2000145 | mod t-test | 910 | 319 | <1.11E-16 |
| regulation of epithelial cell proliferation | GO:0050678 | mod t-test | 326 | 119 | <1.11E-16 |
| regulation of locomotion | GO:0040012 | mod t-test | 948 | 329 | <1.11E-16 |
| regulation of membrane potential | GO:0042391 | mod t-test | 410 | 152 | 3.17E-09 |
| regulation of protein phosphorylation | GO:0001932 | mod t-test | 1046 | 318 | <1.11E-16 |
| regulation of secretion | GO:0051046 | mod t-test | 593 | 210 | <1.11E-16 |
| regulation of secretion by cell | GO:1903530 | mod t-test | 538 | 185 | <1.11E-16 |
| regulation of transport | GO:0051049 | mod t-test | 1646 | 526 | 8.31E-05 |
| regulation of vasculature development | GO:1901342 | mod t-test | 275 | 108 | 9.82E-09 |
| renal system development | GO:0072001 | mod t-test | 292 | 118 | <1.11E-16 |
| renal tubule development | GO:0061326 | mod t-test | 92 | 44 | <1.11E-16 |
| renal tubule morphogenesis | GO:0061333 | mod t-test | 74 | 38 | <1.11E-16 |
| secretion | GO:0046903 | mod t-test | 885 | 310 | <1.11E-16 |
| secretion by cell | GO:0032940 | mod t-test | 750 | 255 | <1.11E-16 |
| tissue development | GO:0009888 | mod t-test | 1757 | 543 | <1.11E-16 |
| transmembrane receptor protein tyrosine kinase signaling pathway | GO:0007169 | mod t-test | 587 | 200 | <1.11E-16 |
| ureteric bud development | GO:0001657 | mod t-test | 90 | 43 | <1.11E-16 |
| urogenital system development | GO:0001655 | mod t-test | 327 | 126 | <1.11E-16 |
| vasculature development | GO:0001944 | mod t-test | 689 | 235 | <1.11E-16 |
| antigen processing and presentation | GO:0019882 | both | 102 | 54 | 2.37E-09 |
| biological adhesion | GO:0022610 | both | 1409 | 580 | 5.90E-14 |
| biological regulation | GO:0065007 | both | 11010 | 2839 | <1.11E-16 |
| cation transport | GO:0006812 | both | 1099 | 390 | 8.78E-16 |
| cell activation | GO:0001775 | both | 957 | 407 | <1.11E-16 |
| cell adhesion | GO:0007155 | both | 1402 | 578 | <1.11E-16 |
| cell communication | GO:0007154 | both | 5704 | 1754 | 1.92E-04 |
| cell killing | GO:0001906 | both | 173 | 79 | 6.80E-15 |
| cell migration | GO:0016477 | both | 1374 | 483 | <1.11E-16 |
| cell population proliferation | GO:0008283 | both | 1838 | 597 | 1.66E-09 |
| cell surface receptor signaling pathway | GO:0007166 | both | 2486 | 850 | <1.11E-16 |
| cell-cell adhesion | GO:0098609 | both | 833 | 360 | <1.11E-16 |
| cellular homeostasis | GO:0019725 | both | 870 | 298 | 7.64E-06 |
| hematopoietic or lymphoid organ development | GO:0048534 | both | 830 | 291 | <1.11E-16 |
| hemopoiesis | GO:0030097 | both | 794 | 278 | <1.11E-16 |
| immune response | GO:0006955 | both | 1581 | 633 | 2.73E-06 |
| immune system development | GO:0002520 | both | 881 | 301 | 6.86E-04 |
| immune system process | GO:0002376 | both | 2350 | 864 | <1.11E-16 |
| intracellular signal transduction | GO:0035556 | both | 2495 | 750 | 9.38E-03 |
| leukocyte mediated cytotoxicity | GO:0001909 | both | 117 | 62 | 7.01E-09 |
| leukocyte proliferation | GO:0070661 | both | 304 | 147 | 3.15E-02 |
| localization | GO:0051179 | both | 5747 | 1548 | <1.11E-16 |
| lymphocyte proliferation | GO:0046651 | both | 276 | 133 | 4.07E-07 |
| mononuclear cell proliferation | GO:0032943 | both | 279 | 135 | 2.76E-03 |
| natural killer cell mediated cytotoxicity | GO:0042267 | both | 64 | 34 | 5.44E-03 |
| negative regulation of cell activation | GO:0050866 | both | 198 | 98 | 1.87E-05 |
| negative regulation of cell adhesion | GO:0007162 | both | 277 | 134 | 2.82E-04 |
| positive regulation of cell adhesion | GO:0045785 | both | 421 | 194 | 3.65E-03 |
| positive regulation of cellular process | GO:0048522 | both | 5219 | 1414 | <1.11E-16 |
| positive regulation of molecular function | GO:0044093 | both | 1434 | 439 | 5.37E-14 |
| regulation of cell activation | GO:0050865 | both | 549 | 252 | <1.11E-16 |
| regulation of cell adhesion | GO:0030155 | both | 712 | 308 | <1.11E-16 |
| regulation of cell population proliferation | GO:0042127 | both | 1573 | 528 | 7.31E-03 |
| regulation of cell-cell adhesion | GO:0022407 | both | 425 | 207 | 2.74E-13 |
| regulation of cellular process | GO:0050794 | both | 9924 | 2545 | <1.11E-16 |
| regulation of hydrolase activity | GO:0051336 | both | 976 | 316 | <1.11E-16 |
| regulation of intracellular signal transduction | GO:1902531 | both | 1592 | 470 | 6.03E-04 |
| regulation of localization | GO:0032879 | both | 2585 | 816 | 3.51E-12 |
| regulation of molecular function | GO:0065009 | both | 2878 | 812 | <1.11E-16 |
| regulation of signal transduction | GO:0009966 | both | 2735 | 801 | <1.11E-16 |
| regulation of signaling | GO:0023051 | both | 3110 | 924 | <1.11E-16 |
| response to cytokine | GO:0034097 | both | 861 | 303 | <1.11E-16 |
| response to hypoxia | GO:0001666 | both | 291 | 107 | <1.11E-16 |
| signal transduction | GO:0007165 | both | 5196 | 1569 | <1.11E-16 |
| signaling | GO:0023052 | both | 5656 | 1745 | <1.11E-16 |
| T cell proliferation | GO:0042098 | both | 191 | 100 | 1.83E-05 |

Supplementary Figure 1. Empirical Cumulative Distribution Function (ECDF) of DE prior scores on differentially expressed genes selected in breast cancer tissues (left panel) and kidney cancer tissues (right panel). A Wilcoxon signed-rank test has been performed to compare the distributions of DE prior scores of genes detected with the Hy-test and the moderated t-test

Supplementary Figure 2a. First two eigenvectors of breast cancer expression genes. Top and bottom panels refer to the first and the second eigenvectors, respectively. Left panels refer to all genes, middle panels refer to the set of genes selected by moderated t-test, and right panels refer to the set of genes selected by hy-test.

Supplementary Figure 2b. Correlation structure of breast cancer expression genes. Left panel refers to the set of genes selected by moderated t-test, and right panel refers to the set of genes selected by hy-test. The correlation structures are obtained by ordering the genes according to the scores of the second principal component (bottom panels of Figure 1a).

Supplementary Figure 3a. First two eigenvectors of kidney cancer expression genes. Top and bottom panels refer to the first and the second eigenvectors, respectively. Left panels refer to all genes, middle panels refer to the set of genes selected by moderated t-test, and right panels refer to the set of genes selected by hy-test.

Supplementary Figure 3b. Correlation structure of kidney cancer expression genes. Left panel refers to the set of genes selected by moderated t-test, and right panel refers to the set of genes selected by hy-test. The correlation structures are obtained by ordering the genes according to the scores of the second principal component (bottom panels of Figure 1a).
